# Dose-dependent galactorrhea with quetiapine

**DOI:** 10.4103/0019-5545.74315

**Published:** 2010

**Authors:** Sujata Sethi, Manisha Sharma, Amit Malik

**Affiliations:** Department of Psychiatry, Pt. BD Sharma University of Health Sciences, Rohtak, Haryana, India

**Keywords:** Galactorrhea, hyperprolactinemia, quetiapine

## Abstract

Quetiapine is an atypical antipsychotic agent with minimal propensity to induce hyperprolactinemia in standard therapeutic dosages. Despite that quetiapine is considered to be a prolactin-sparing atypical antipsychotic, hyperprolactinemia with related side effects may rarely be encountered in susceptible individuals. We report a case of quetiapine-induced hyperprolactinemia and galactorrhea in an adult female, which was dose-dependent.

## INTRODUCTION

Galactorrhea is an embarrassing side effect of antipsychotic drugs, especially with the typical ones. Among the atypical antipsychotic drugs quetiapine is the least galactogenic and has been recommended as an alternative in cases that develop galactorrhea with other antipsychotics.[1–3] Unlikely though it is, we report a case of quetiapine-induced galactorrhea that was dose-dependent.

## CASE REPORT

Mrs. DC, a 40-year-old woman, with a nine-year history of bipolar affective disorder, experienced repeated episodes of both depression and mania over the years. Prophylactic treatment with lithium carbonate and sodium valproate was not successful and she developed extrapyramidal side effects readily with risperidone. Olanzapine also had to be discontinued as she put on weight rapidly. During one of the manic episodes, quetiapine was started both for therapeutic as well as prophylactic purposes. The dose was titrated to 400 mg daily over a period of three weeks. She showed good response, but after two months of continued treatment she complained of amenorrhea and galactorrhea. Serum prolactin estimation showed raised prolactin levels (45.5 ng/mL; normal range 2.80 – 29.20 ng/mL).

Pregnancy test was negative and a normal thyroid function test excluded hypothyroidism from being the cause of galactorrhea. A brain scan was not performed as the serum prolactin elevation was less than twice the upper limit of normal.

As quetiapine was assumed to be the culprit, the dose of quetiapine was reduced to 250 mg per day over the next two weeks. The galactorrhea subsided in the next week. She, however, took another two months to resume her normal menstrual cycle. A repeat serum prolactin estimation at this stage showed normal levels (11.89 ng/mL). Mrs. DC continued to keep well on the reduced dose of quetiapine.

## DISCUSSION

Quetiapine is an atypical antipsychotic drug with a very low propensity to induce hyperprolactinemia in the standard, recommended, therapeutic doses. This prolactin sparing property of quetiapine and other atypical agents is due to the lesser occupancy of DA D_2_ receptors on lactotrope cells in the anterior pituitary gland, as compared to the striatal DA D_2_ occupancy.[4]

Yet, there have been reports of its adverse effects on prolactin. Alexiadis *et al*.[5] and Stevens *et al*.[6] have reported elevation of prolactin levels due to quetiapine, but with no subsequent galactorrhea and/or amenorrhea. Gupta[7] has reported quetiapine-induced galactorrhea in a female patient, but the patient was taking other psychotropics also, including venlafaxine, a drug that affects the dopamine system as well. Furthermore, galactorrhea appeared at a very low dose of quetiapine (100 mg) within 10 days of treatment. To the best of our knowledge there is only one case report by Buhagiar and Cassar[8] reporting dose-dependent galactorrhea with quetiapine in an adolescent boy, and none in adults.

Despite that quetiapine is considered to be a prolactin-sparing atypical antipsychotic, hyperprolactinemia with related side effects may rarely be encountered in susceptible individuals. Similar dose-dependent elevation of serum prolactin with quetiapine treatment has been reported by Stevens *et al*.[6] However, these side effects seem to subside with reduction in the dosage of quetiapine. Therefore, it is worthwhile reducing the dose rather than switching to another antipsychotic, considering the high safety and tolerability profile of the drug.
